# Women's studies in the Latin American context: a bibliometric approach

**DOI:** 10.12688/f1000research.159117.1

**Published:** 2025-02-26

**Authors:** Jackeline Valencia, Ada Gallegos, Jeri Gloria Ramón Ruffner, Ezequiel Martínez-Rojas, Alejandro Valencia-Arias, Martha Luz Benjumea Arias, Gina Ore León, Lucia Palacios-Moya

**Affiliations:** 1 Instituto de Investigación y Estudios de la Mujer, Universidad Ricardo Palma, Santiago de Surco, Lima, 15039, Peru; 2 Universidad Nacional Mayor de San Marcos, Lima District, Lima Region, 15081, Peru; 3 Vicerrectoría de Investigación e Innovación, Universidad Arturo Prat, Iquique, Tarapacá Region, 1110939, Chile; 4 Facultad de Ciencias Económicas y Administrativas, Instituto Tecnologico Metropolitano, Medellín, Antioquia, 50010, Colombia; 5 Universidad de San Martin de Porres Facultad de Medicina Humana, La Molina, Lima Region, 15039, Peru; 6 Escuela de posgrado, Universidad Senor de Sipan, Chiclayo, Lambayeque, Peru

**Keywords:** Gender inequality, Just society, Gender violence, Female entrepreneurship, Neoliberalism, Female empowerment, Political participation, PRISMA-2020.

## Abstract

**Background:**

Women’s studies in Latin America are transcendental because they make visible and challenge gender inequalities to achieve a more just and equitable society. However, despite this, there are still many research gaps, for which the objective is to examine the trends in research on women’s studies in Latin America.

**Methods:**

An exploratory methodology based on bibliometric analysis is proposed to evaluate the scientific literature, based on the parameters of the PRISMA-2020 declaration.

**Results:**

The bibliometric analysis reveals a growth in women’s studies in Latin America, reflecting its importance and relevance. Scientific production has experienced exponential growth, with leading researchers and journals. The United States and Canada lead scientific production. A change is observed in the topics addressed, focusing more on gender and equality. The thematic clusters identified highlight priority areas such as politics, institutions and representation.

**Conclusions:**

Emerging keywords include neoliberalism, gender violence, political participation, female empowerment, and femicide, reflecting new concerns and challenges addressed in gender studies in Latin America.

## 1. Introduction

### 1.1 Subtitle

Women’s studies in Latin America is a constantly growing field of research that seeks to understand and address the various dimensions of gender inequality in the region. These studies focus on analyzing the experiences, social roles, cultural practices, public policies and empowerment processes of Latin American women. Through the analysis of these issues, it seeks to generate knowledge that contributes to the promotion of gender equality and the improvement of the living conditions of women in the region.

There is a relationship between gender inequality, women’s empowerment and adolescent pregnancy rates in Latin American cities ( Braverman-Bronstein et al., 2023). Her research shows the importance of addressing gender inequality as a key variable in understanding and addressing the high rates of teen pregnancy in the region. In addition, another study highlights the importance of public service campaigns on violence against women in Latin America ( Mensa and Grow, 2023). Their study analyzes how these campaigns can contribute to the awareness and prevention of gender violence in the region. The work highlights the crucial role of education and public awareness to face this serious social problem.

On the other hand, the empowerment of women and the fight against violence in Latin America are addressed. This research highlights the importance of strengthening the political and social participation of women as a means to combat gender violence ( Sanín, 2023). The article proposes strategies and recommendations to promote gender equality and the security of women in the region. Regarding violence against women in politics, the process of criminalization of this violence is explored, analyzing its innovation, diffusion and transformation ( Sanín, 2022). The study highlights the challenges and advances in the protection of women politicians against gender violence, and highlights the importance of addressing this problem from a legal and political perspective.

The following article focuses on the role of women in the continuity of family businesses in rural areas of Honduras. Research shows the significant contribution of women as entrepreneurs and managers in the sustainability and success of family businesses in rural settings ( Cruz et al., 2022). The study highlights the importance of recognizing and valuing the participation of women in the rural economy.

These investigations have become a fundamental tool to understand and address the challenges faced by women in the region, as well as to promote gender equality and female empowerment. In this sense, the need to analyze the role of women in the academy and the care practices they perform is highlighted ( Castelao-Huerta, 2023). These studies allow us to understand how women academics face specific challenges and how policies and institutional changes can be implemented to guarantee equal opportunities and gender inclusion in the academic field.

In turn, this article highlights the contribution of Argentine women to the knowledge of India in Latin America, highlighting the importance of recognizing and making visible the contributions of women in the generation of knowledge and in the construction of international academic networks ( Canzobre, 2022). While, in this other article, the research explores the challenges faced by women in politics and the double glass ceiling they must overcome ( Kouba and Dosek, 2023). These studies are essential to understand the barriers and obstacles that prevent the equal representation of women in political processes and to promote the political participation of women in the region.

However, there are still important research gaps that justify carrying out exhaustive bibliometrics in this area. For example, in the following article they systematically mapped the literature on Latin American women in computing, highlighting the scarcity of studies that address the specific barriers and challenges they face in this discipline ( Holanda & Da Silva, 2021). In addition, a systematic review of the literature on the role of women in the care economy during the COVID-19 pandemic in Latin America was carried out, revealing the need for further research in this crucial field ( Malaver-Fonseca et al., 2021). These investigations underscore the importance of bibliometrics in women’s studies in Latin America, since it would allow the identification of existing knowledge gaps, as well as emerging areas of research that require more attention to promote gender equality and the empowerment of women in Latin America. the region. Therefore, the objective is to examine the trends in research on women’s studies in Latin America, so that, based on this, a research agenda for future studies can be structured, for which, in addition, there are the following research questions:

• What are the years where more interest has been presented in women’s studies in Latin America?
• What kind of growth does the number of scientific articles on women’s studies in Latin America show?
• What are the main research references on women’s studies in Latin America?
• What is the thematic evolution derived from the scientific production on women’s studies in Latin America?
• What are the main thematic clusters on women’s studies in Latin America?
• What are the main clusters of scientific associativity in research on women’s studies in Latin America?
• What are the growing and emerging keywords in the field of women’s studies research in Latin America?

The article is structured in such a way that it begins with a review of the relevant literature in the field of women’s studies in Latin America. Then, in the methodological section, the study design and the methods used to collect and analyze the data are described in detail, in order to answer the questions posed previously. The findings are presented in the results section, where relevant data and statistics are included, and finally, the results are interpreted and their implications are discussed in the final section of the article.

## 2. Methods

In order to meet the proposed research objective, an exploratory scope methodology based on secondary sources of information is proposed through a bibliometric analysis that allows an evaluation of the scientific literature on the study of women in Latin America, to this follows the parameters of the PRISMA-2020 international declaration ( Page et al., 2021).

### 2.1 Eligibility criteria

The inclusion criteria for this research are defined by articles that explicitly include keywords such as “women” and “Latin America” in their titles, which serve as primary scholarly metadata. This deliberate choice is consistent with the specificity of the research and the diverse terminology used in the scholarly literature. The inclusion criteria are designed to account for the multiple ways in which relevant studies may refer to the topic. A careful three-step process then governs the exclusion criteria. The first stage systematically eliminates records with erroneous indexing, a crucial step to ensure the accuracy and reliability of the results.

The second stage involves the removal of documents without accessible full text, focusing primarily on systematic reviews, as bibliometric analysis prioritizes the examination of metadata rather than comprehensive textual content. The third and final stage of exclusion removes conference proceedings, documents with incomplete indexing, and texts deemed of marginal relevance to the study. The meticulous application of these inclusion and exclusion criteria is crucial for a precise and rigorous selection of articles, which is essential for the subsequent bibliometric analysis of women’s studies in Latin America.

This rigorous selection process not only ensures the relevance and accuracy of the articles identified, but also serves to streamline the subsequent bibliometric analysis. By strategically incorporating these criteria, the research aims to create a focused dataset that aligns precisely with the overarching objectives of the study. This systematic approach increases the reliability of the bibliometric analysis and ensures that the selected articles are truly representative of the diverse landscape of women’s studies in the Latin American context.

### 2.2 Source of information

To carry out the source of information with a bibliometric study on women’s studies in Latin America, the Scopus and Web of Science databases have been selected, which are recognized as the main sources of scientific information today. Scopus is a multidisciplinary database that covers various areas of knowledge, while Web of Science focuses mainly on the natural and social sciences. Both databases offer a wide coverage of scientific journals and conferences, which allows obtaining a complete picture of the scientific production related to the topic of interest. In addition, both databases are widely used and recognized in the academic field, which guarantees the quality and reliability of the data obtained. A relevant study that highlights the importance of combining data from Scopus and Web of Science in bibliometric analysis, the following authors present a simple and useful method to merge data from both databases during bibliometric analysis ( Caputo and Kargina, 2022).

### 2.3 Search strategy

To carry out the search strategy in the databases selected for bibliometrics on women’s studies in Latin America, two specialized search equations were designed, which were adapted to the inclusion criteria and the search characteristics of each database. In the case of the Scopus database, the search equation is the following:

For the Scopus database: ((TITLE (wom?n) AND TITLE (“Latin America” OR “Latin-America” OR “Latinamerica”)) OR (AUTHKEY (wom?n) AND AUTHKEY (“Latin America” OR “Latin-America” OR “Latinamerica”))) AND (EXCLUDE (SUBJAREA, “MEDI”) OR EXCLUDE (SUBJAREA, “EART”) OR EXCLUDE (SUBJAREA, “NURS”) OR EXCLUDE (SUBJAREA, “BIOC”) OR EXCLUDE (SUBJAREA, “AGRI”) OR EXCLUDE (SUBJAREA, “IMMU”) OR EXCLUDE (SUBJAREA, “ENER”) OR EXCLUDE (SUBJAREA, “CHEM”) OR EXCLUDE (SUBJAREA, “CENG”) OR EXCLUDE (SUBJAREA, “HEAL”) OR EXCLUDE (SUBJAREA, “GO”) OR EXCLUDE (SUBJAREA, “MATE”) OR EXCLUDE (SUBJAREA, “NEUR”) OR EXCLUDE (SUBJAREA, “PHAR”))

For the Web of Science database: ((TI= (wom?n) AND TI = (“Latin America” OR “Latin-America” OR “Latinamerica”)))

It is important to note that both specialized equations are similar, but the modifications applied in terms of country and research area filters are reflected only in the Scopus database equation, while for the Web of Science equation, the Filters are only displayed in the platform and not in the equation itself.

### 2.4 Data management

In the study of bibliometrics on women’s studies in Latin America, the Microsoft Excel ^®^ tool was used to extract, store, and process the information from each analyzed database. In addition, the free software VOSviewer ^®^, in its version 1.6.18, was used in conjunction with Microsoft Excel ^®^ to generate graphic representations of the different bibliometric indicators obtained. VOSviewer ^®^ is a network analysis and visualization tool that allows you to explore and visualize the structure of scientific publications. To carry out this research, the APA-style bibliographic reference proposed by several authors who describe the use of VOSviewer in combination with bibliometrics ( Arruda et al., 2022) was followed.

### 2.5 Selection process

According to the PRISMA 2020 statement, highlighted in the article by Page et al. (2021), it is key to mention whether an internal automatic classifier was used to support the selection process, as well as internal or external validation in order to understand the risk of omitted studies or incorrect classifications. In this particular study, Microsoft Excel ^®^ automation tools were used as an internal resource. These tools were developed by all study investigators, who in turn used them independently to apply the inclusion and exclusion criteria. This was done with the aim of minimizing the risk of omitting relevant studies or misclassifying through convergence of the results obtained.

### 2.6 Data collection process

In the data collection process, a bibliometric on women’s studies in Latin America was carried out, following the guidelines proposed by Page et al. (2021). To collect the data for the reports, specific methods were used. Each report was reviewed by all study authors, who served as reviewers independently. This confirmed a rigorous validation of the data obtained. In addition, Microsoft Excel ^®^ was used as an automated tool for the data collection process from the two selected databases. This tool facilitated the organization and systematization of the information collected. Subsequently, a collective data confirmation process was carried out, in which all the authors collaborated to achieve absolute convergence in the results. This approach guaranteed the accuracy and reliability of the data collected in the bibliometric study on women’s studies in Latin America.

### 2.7 Data elements

To carry out the data elements of the bibliometrics on women’s studies in Latin America, specific data searches were carried out in each article. These data include the results obtained in each study, such as measurements, time points, and analyzes performed. We sought to collect all the results compatible with the research objective, using a specialized search equation designed for each database. However, if there was missing or unclear information in any article, it was excluded considering it as irrelevant text, since it did not contribute to understanding the knowledge base on the subject. This was done to ensure consistency with the purpose and scope of the investigation.

### 2.8 Study risk of bias assessment

In the context of this bibliometric review of women’s studies in Latin America, it is imperative to describe the methods used to assess the risk of bias inherent in the studies included. The term “risk of bias” refers to the potential distortion of study results due to systemic flaws in their design, conduct, or analysis. In particular, the entire cohort of authors involved in this review conducted the data collection process. To ensure the quality and integrity of the results obtained, a risk of bias assessment was meticulously conducted through a four-day analysis using Microsoft Excel ^®^, a widely used scientific data analysis application.

By choosing an automated tool to assess risk of bias, our goal was to mitigate potential bias due to individual interpretation or reviewer subjectivity. In addition, this approach promotes greater consistency and comparability in the assessment of studies embedded in bibliometrics. It is important to note that the assessment process took into account both the number of reviewers involved in each study and whether they acted independently. These considerations contribute significantly to the reliability of the results, as they introduce multiple perspectives and help to avoid possible individual influences or biases. Importantly, the expedited four-day analysis was instrumental in avoiding prolonged exposure to the data, thereby minimizing the risk of unintentional bias.

### 2.9 Measures of effect

Bibliometrics is a methodology used to analyze and evaluate scientific production in a given field of study. In the case of research on women’s studies in Latin America, it is important to specify the effect measures used in the synthesis or presentation of the results. These effect measures, such as the risk ratio or the mean difference, are commonly used in primary research to quantify the impact of an intervention or variable on a specific outcome.

However, in this particular investigation, secondary research sources are used, which implies that primary data are not available to calculate these measures of effect directly. Instead, a bibliometric analysis is carried out using Microsoft Excel ^®^ to examine the number of publications and the number of citations related to women’s studies in Latin America. This allows us to have an overview of the scientific production in this field and to evaluate its impact in the academic community.

In addition, VOSviewer ^®^ is used to determine the thematic association between the publications and the keywords used in them. This software allows you to visualize the existing nodes and analyze the relationship and grouping of the key terms. By looking at the timing of each keyword’s usage, trends and changes in research can be identified over time.

### 2.10 Synthesis methods

In the present study on women’s studies in Latin America, several processes were used to determine which studies were eligible for inclusion in the synthesis. These processes included the tabulation of the intervention characteristics of each study and their comparison with the groups planned for each synthesis. In addition, methods were used to prepare the data prior to presentation or synthesis, which involved handling missing summary statistics and necessary data conversions. To tabulate or visually display the results of individual studies and syntheses, specific methods were used.

In this study, bibliometric indicators of quantity, quality and structure were also carried out ( Durieux and Gevenois, 2010), which were applied automatically using Microsoft Excel ^®^ to all those documents that passed the three exclusion phases. In this way, it was possible to obtain a quantitative and qualitative evaluation of women’s studies in Latin America, allowing a more complete and rigorous analysis of the subject.

### 2.11 Assessment of reporting bias

In this study, the possibility of a bias towards certain synonyms found in thesauri such as the IEEE is recognized, which may manifest itself in the inclusion criteria, search strategy and data collection. In addition, since these are conference proceedings, documents with incomplete indexing and irrelevant texts as exclusion criteria, it may result in the omission of valuable information for the construction of knowledge on the subject. Therefore, it is critical to approach these methodological challenges with caution and seek complementary approaches to minimize the risk of bias in bibliometrics results.

### 2.12 Certainty assessment

**Figure 1.**
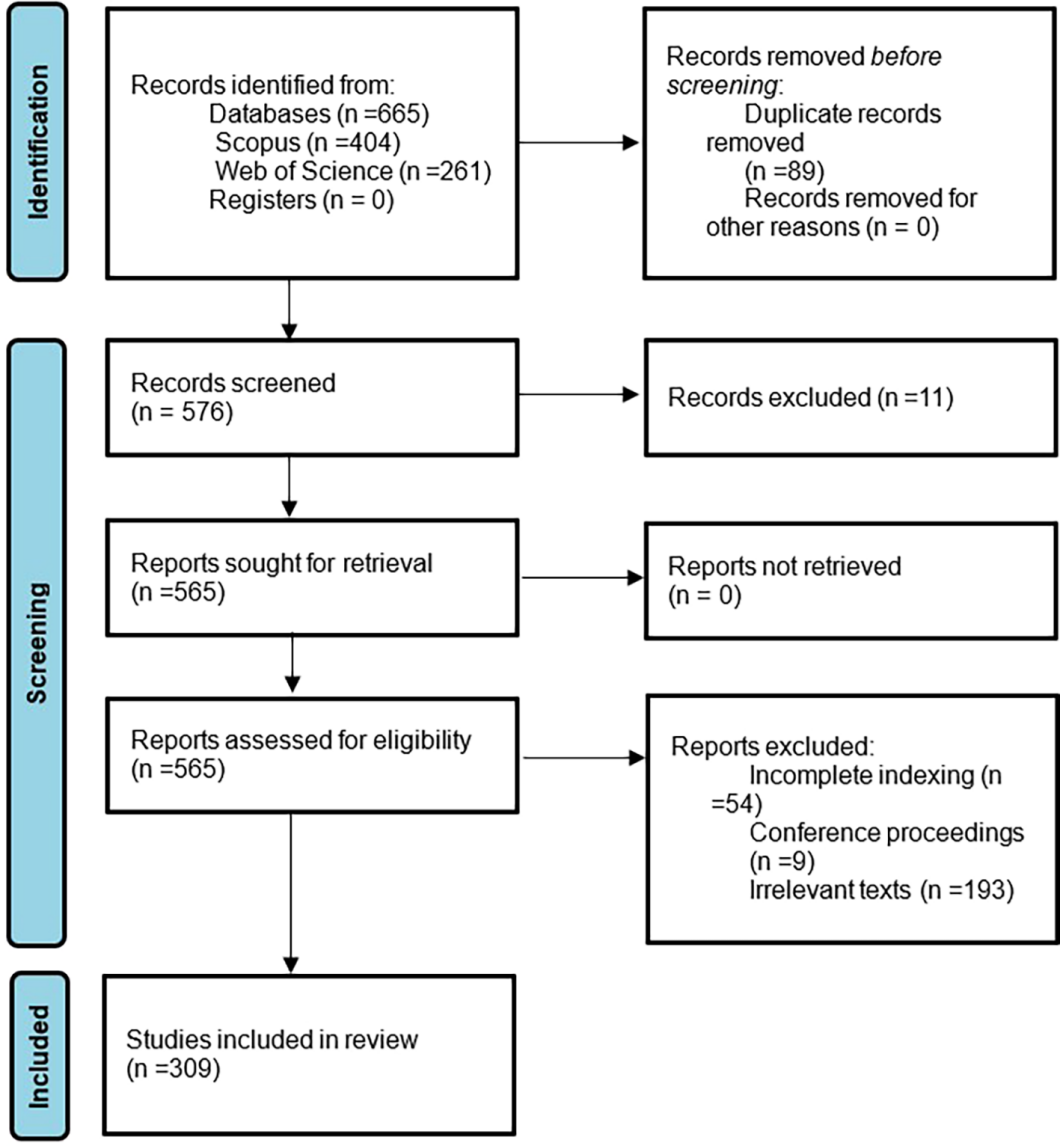
PRISMA flow chart. Own elaboration from Scopus and Web of Science.

In this bibliometric investigation of women’s studies in Latin America, an approach is used to assess the certainty or confidence in the body of evidence for a result. Unlike primary studies that assess certainty individually, in this case it is assessed in a general way through the independent application of inclusion and exclusion criteria, as well as the definition of bibliometric indicators. In addition, possible defined biases in the methodological design are reported, and limitations of the study are mentioned in the discussion phase. This approach allows us to have a global vision of the certainty in the body of evidence and highlight its strengths and weaknesses. Subsequently, to summarize the methodological design, there is the following flowchart which is in Figure 1.

**Figure 2.**
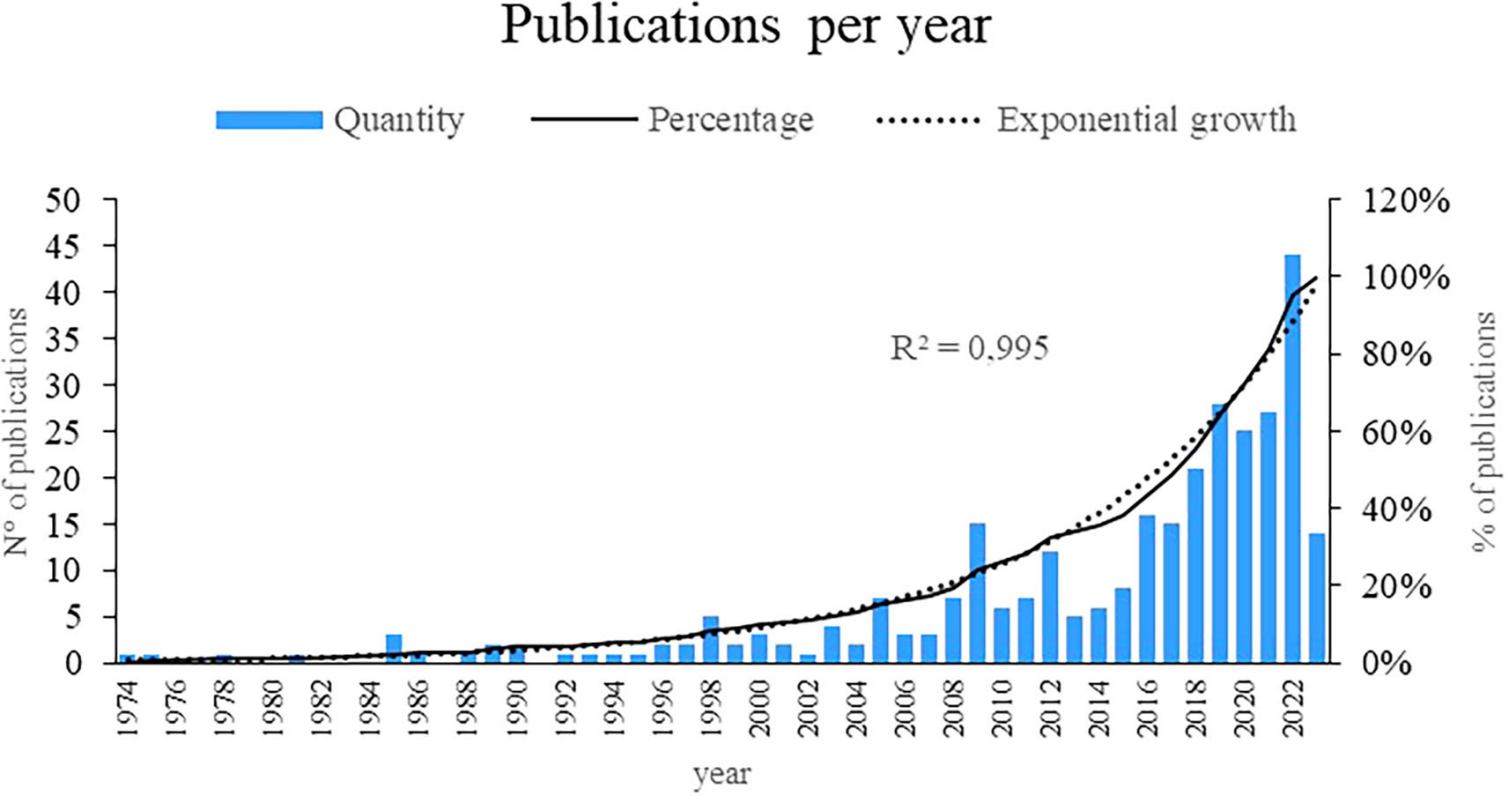
Publications by year. Own elaboration from Scopus and Web of Science.

The initial phase of identification of articles resulting from the application of the search strategy for each source of information is identified, as well as the exclusion of all duplicate documents, subsequently there are the three phases or consecutive stages of exclusion defined in the eligibility criteria. and finally, there are the 309 articles, between 1974 and 2023, that will be analyzed in the present bibliometrics on women’s studies in Latin America.

## 3. Results

Initially, the present bibliometric analysis revealed an exponential growth of 99.5% in the annual production of scientific articles on women’s studies in Latin America, with 2019, 2020, 2021 and 2022 being the years with the highest number of publications. related to the subject, as evidenced in Figure 1, revealing a growing interest and recognition of the importance of research in recent decades, particularly in recent years, indicating greater attention and commitment to academic production in this field.

**Figure 3.**
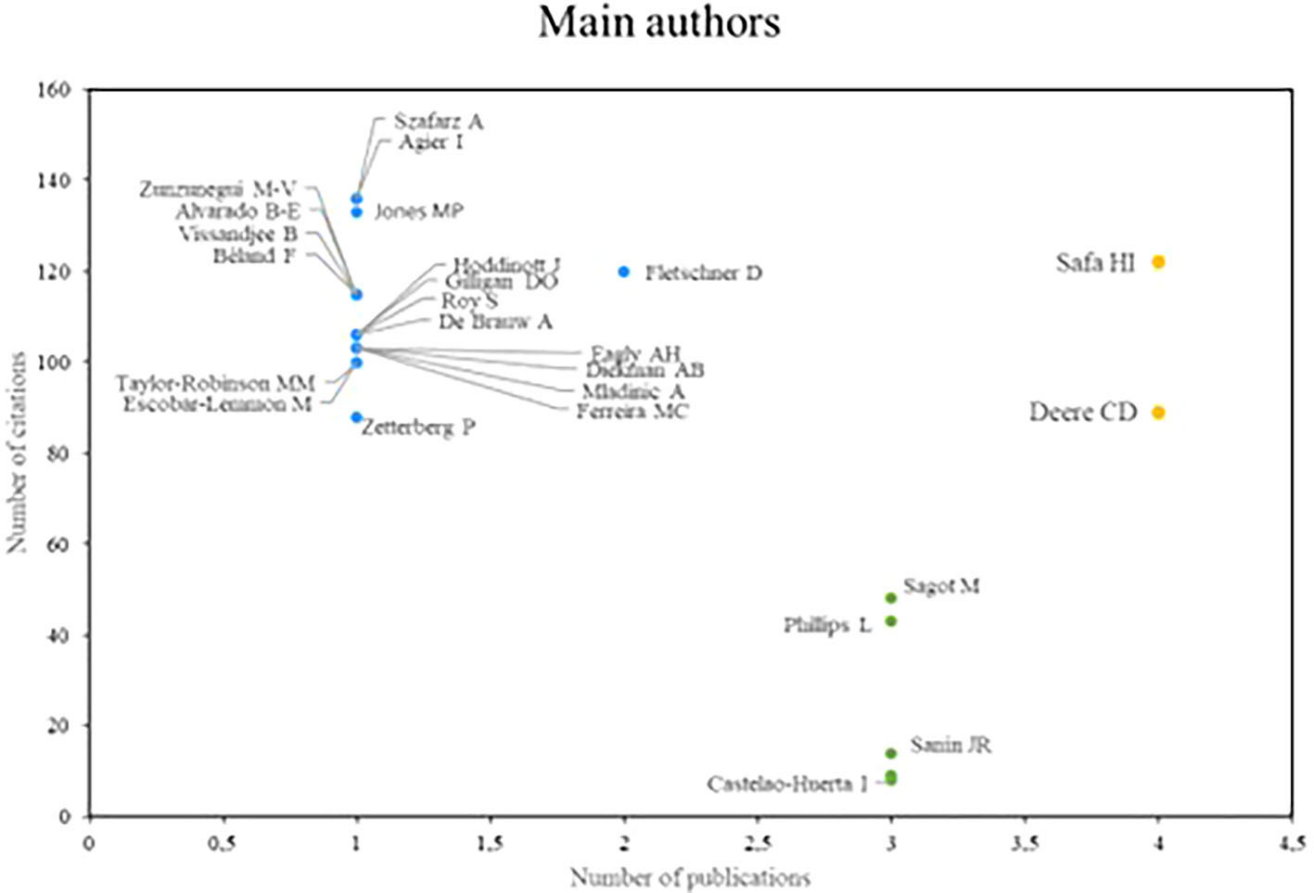
Main authors. Own elaboration from Scopus and Web of Science.

Regarding the research referents related to women’s studies in Latin America, Figure 3 analyzes the authors who stand out the most in productivity, which is measured by the number of publications and academic impact, which is determined by the number of citations that authors receive. Based on the above, three groups of prominent authors were identified. In the first place, there are the authors who stand out both in number of publications and in the number of times they have been cited; in this case, Safa and Deere are research references for scientific production in relation to the subject.

The authors who stand out for their impact on research, despite having a low productivity index, make up the second group, as is the case of Szafarz and Agier, Jones and Fletschner. Finally, in the third group are the authors Sagot and Phillips, who stand out mainly for their high scientific productivity, however, they do not have a significant number of citations.

**Figure 4.**
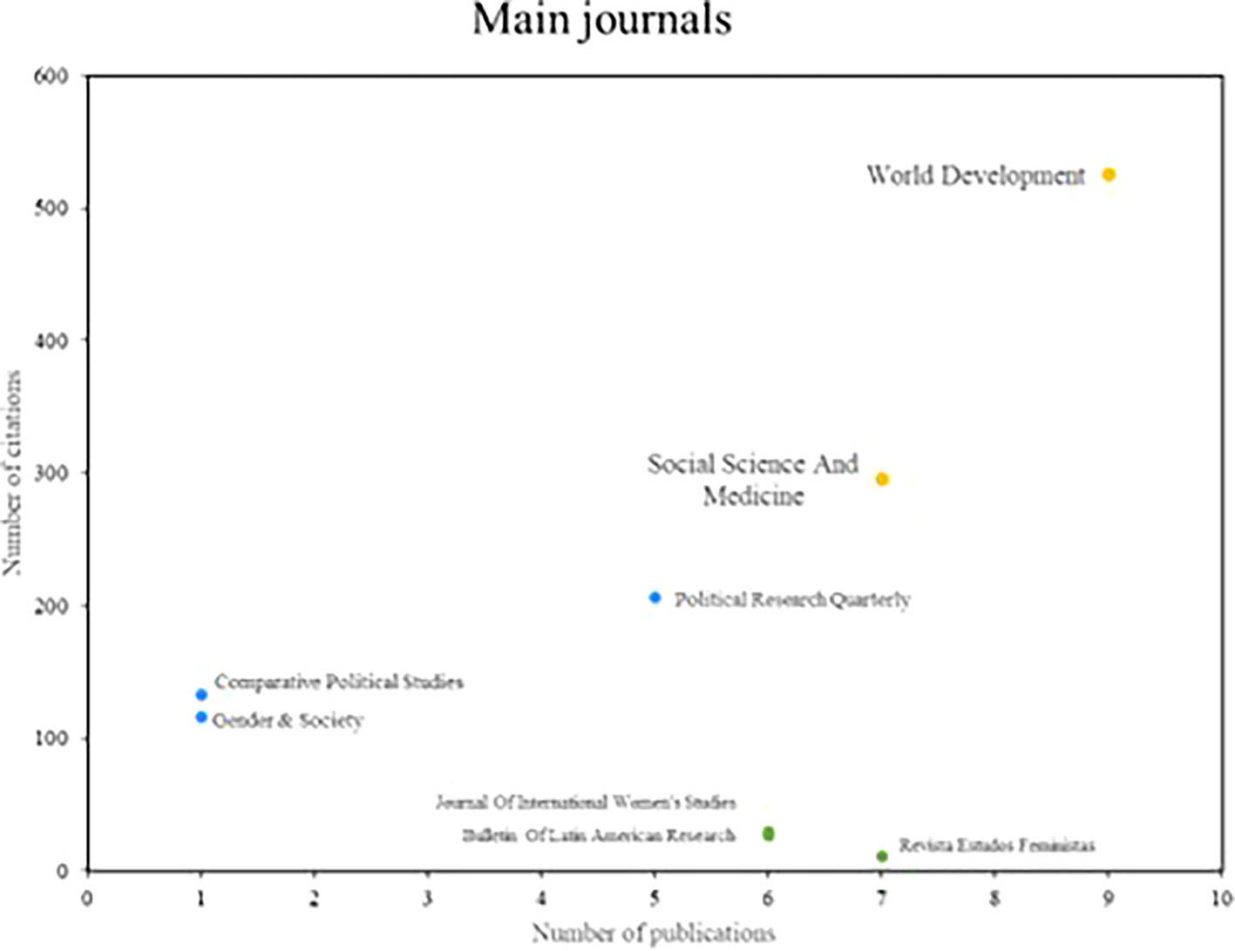
Main journals. Own elaboration from Scopus and Web of Science.

Below, Figure 4 shows the main popular science journals, which are benchmarks in number of publications and number of cumulative citations in research on women’s studies in Latin America. These magazines are divided into three distinct segments. In the first group are those journals that stand out in terms of productivity and impact, such as World Development and Social Science and Medicine, which have 7 or more publications and a high number of citations, positioning them as benchmarks in the field of study. Subsequently, there is the second group where journals such as Political Research Quarterly and Comparative Political Studies are located, which are positioned as benchmarks in terms of printing, each having more than 100 citations, despite having low scientific productivity. On the other hand, the Revista Estados Feministas is part of the third group of scientific journals that are distinguished by their high productivity, but do not stand out for the number of citations received.

**Figure 5.**
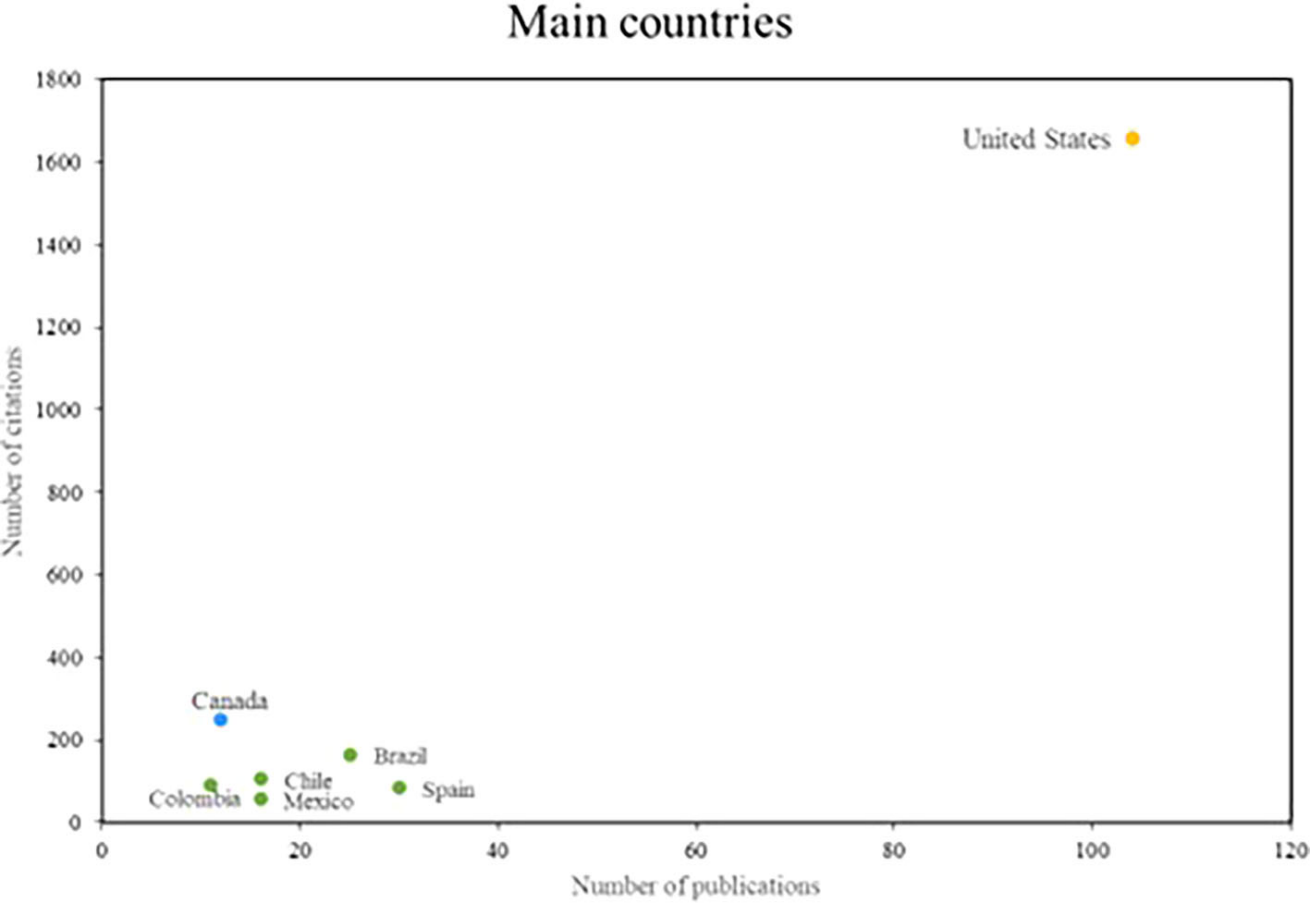
Main countries. Own elaboration from Scopus and Web of Science.

Finally, in relation to the research references, the main countries of scientific production on women’s studies in Latin America are recognized; where three groups of outstanding countries were identified, shown in Figure 5. The United States is the country that stands out the most in the number of publications, with more than 100 publications and more than 1600 citations. On the other hand, in the second group of countries that stand out for their research impact, but not for their scientific productivity; with more than 10 publications and more than 200 citations, only Canada is part of this group. Finally, the countries that stand out for their scientific productivity, but do not have a high number of citations, are recognized. Spain and Brazil are the main references for this group.

**Figure 6.**
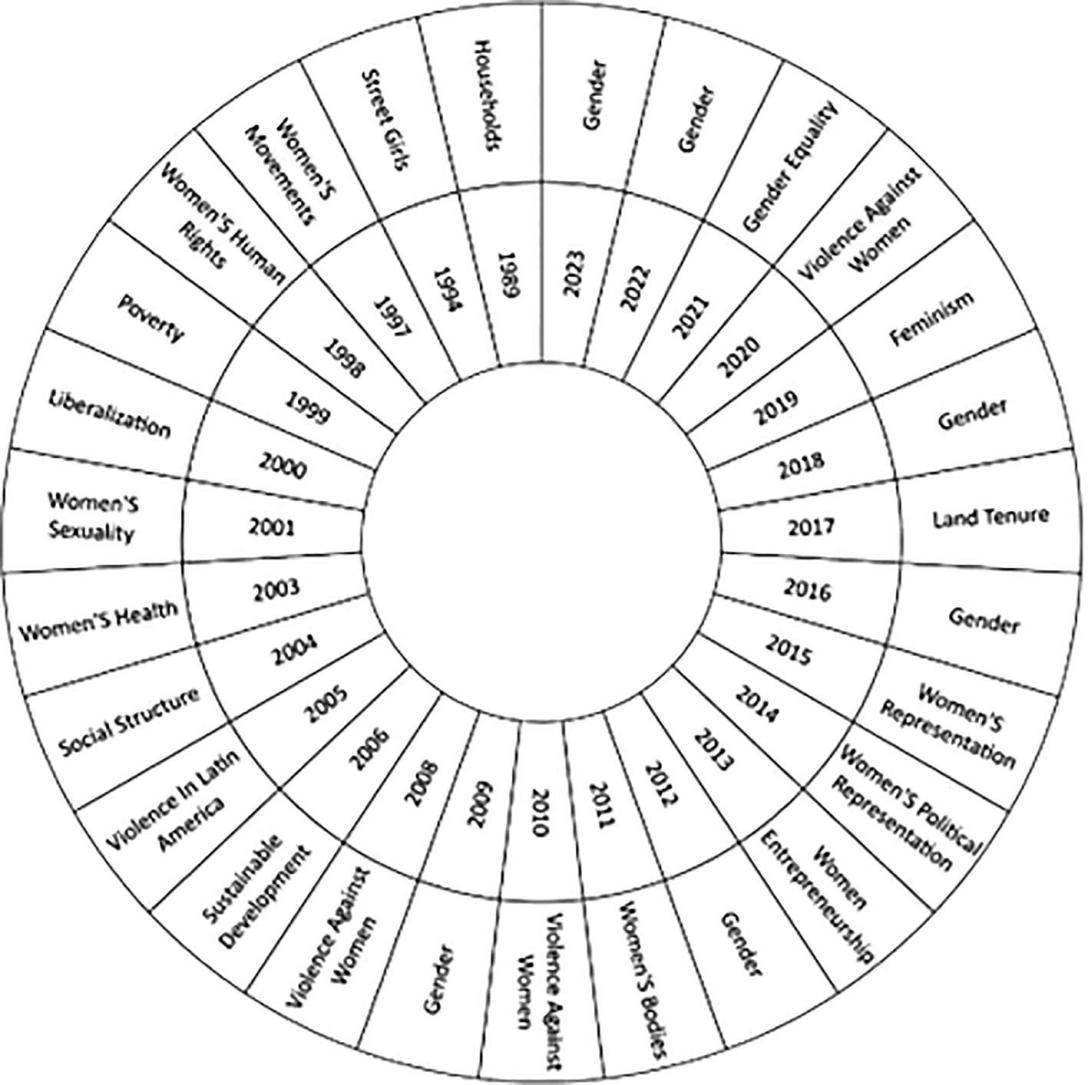
Thematic evolution. Own elaboration from Scopus and Web of Science.

On the other hand, the present study in Figure 6, has the purpose of recognizing the changes and trends in the theme over time, studying the thematic evolution in the literature in relation to the studies of women in Latin America throughout over the years, based on the most used keywords in each year of research; providing an overview of the evolution and changes in the areas of study, as well as the most outstanding topics in the scientific literature. It is important to highlight that the first year considered in the analysis is 1989, the appearance of concepts such as Households is observed, while as time progresses issues such as Gender, Gender Equality, Violence Against Women and Feminism stand out, which reflects the most recent research trends on the subject in the scientific literature.

**Figure 7.**
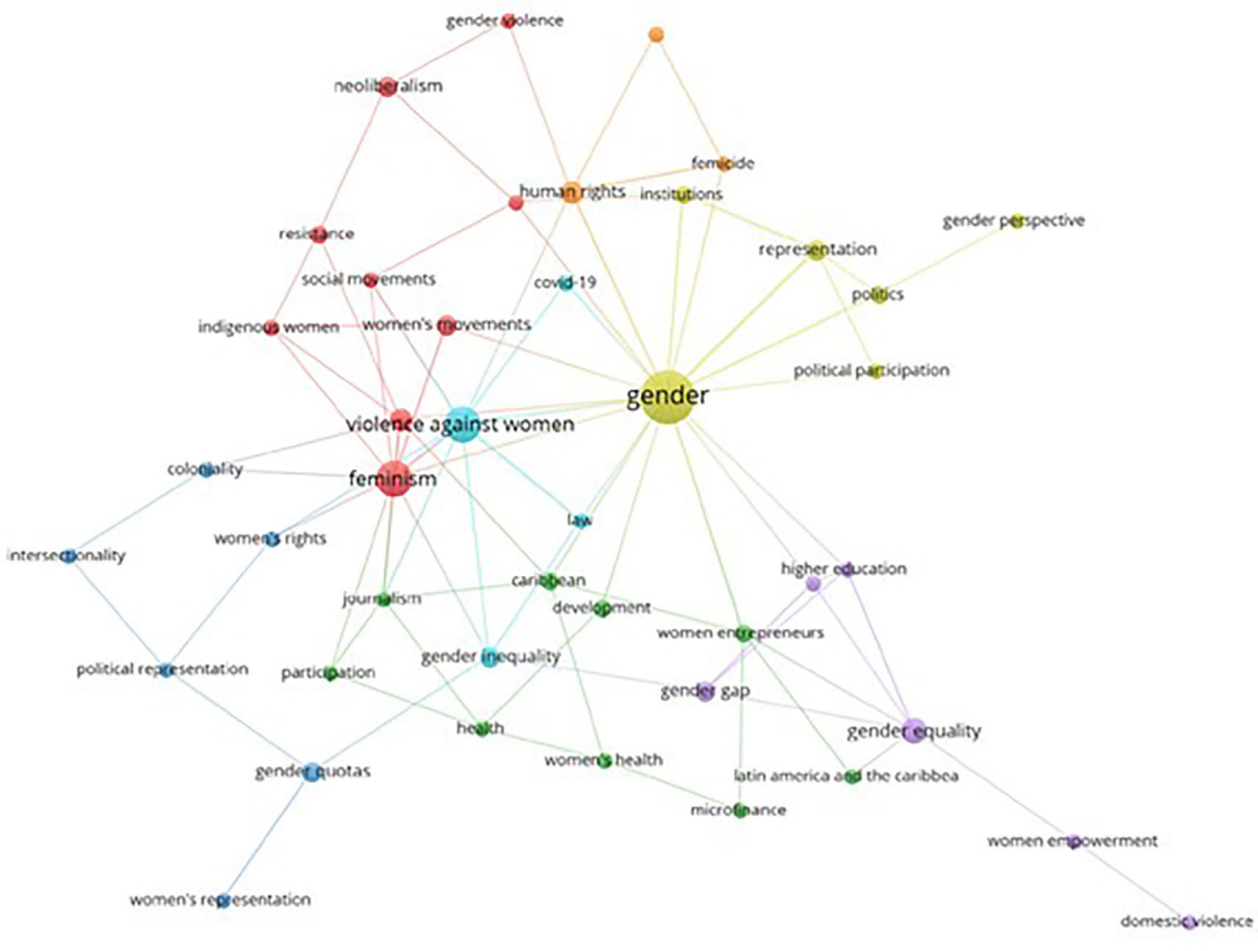
Keyword co-occurrence network. Own elaboration from Scopus and Web of Science.

Based on the keywords registered in each scientific publication, this bibliometrics analyzes the co-occurrence network of the keywords found in the articles related to women’s studies in Latin America, as evidenced in Figure 7, represented through 7 thematic clusters. The yellow cluster stands out for including terms such as Gender, Political Participation, Institutions, Gender Perspective, Politics and Representation. Following this in second place is the red cluster that stands out for the number of concepts that make it up, such as Feminism, Violence, Women’s Movements, Indigenous Women, Social Movements, Resistance, Neoliberalism and Gender Violence. In addition, additional clusters of orange, blue, green and violet colors are identified that represent other elements of conceptual affinity.

**Figure 8.**
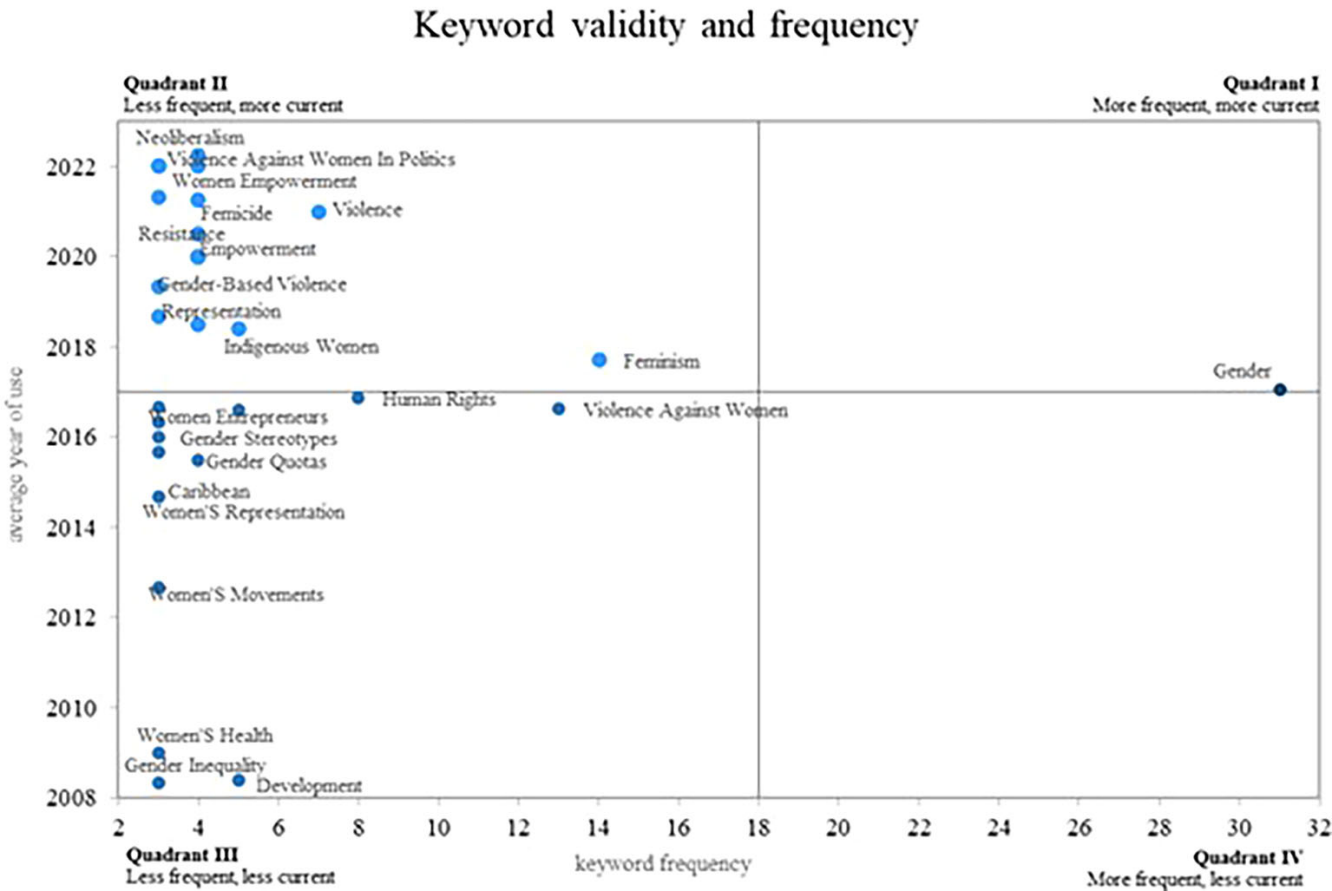
Validity and frequency of the keywords. Own elaboration from Scopus and Web of Science.

Finally, in relation to the results, Figure 8 provides a Cartesian plane, where the frequency of use of each of the keywords is compared, with the validity that each of these have, which is determined by the year. Average use that each one has in the scientific literature, in this sense four different quadrants are displayed.

Initially, in quadrant 4 are those terms that have decreased in the most recent years, although they were not found for this quadrant. Subsequently, in quadrant 2 are the keywords that have a low frequency of use; however, they remain valid, which positions them as emerging concepts. Some of these words are Neoliberalism, Violence Against, Women in Politics, Women Empowerment and Femicide. Additionally, in quadrant 1 are the concepts that are frequently used by the authors, these being considered as consolidated and growing terms, as is the case of the gender concept, since its average year of use is recent, thus confirming its importance today.

## 4. Discussion and Conclusions

In terms of discussion of the results, this section will discuss in depth aspects associated with the growth of scientific literature, the main referents, thematic evolution, thematic clusters and the frequency and validity of keywords, as well as other findings derived from the results, such as the classification of keywords according to their function, the research gaps identified, as well as the limitations of the study and its practical implications, to finally present the main research agenda on women’s studies.

### 4.1 Discussion of results

This bibliometric analysis reveals a remarkable growth in the annual scientific production on women’s studies in Latin America, especially between the years 2019 and 2022. These results reflect a significant increase in interest and recognition of the importance of research in this area in recent decades. The literature reviewed supports this trend, showing an increase in publications on different topics related to women’s experiences in the region.

A noteworthy aspect of the discussion is the diversity of topics addressed in different years. In 2019, the focus was on issues such as gender-based violence, especially against transgender women, highlighting the need for a multidisciplinary approach to address this issue ( Lanham et al., 2019). In 2020, the relationship between corruption and innovation in the private sector and political violence against women was an area of research, highlighting the importance of considering gender in this type of analysis ( Wellalage et al., 2020; Sanín, 2020).

The year 2021 stands out for research on public dissatisfaction with legislative nominations and the review of literature on the role of women in the care economy during the COVID-19 pandemic. These studies link to existing literature on women’s political representation and the valuation of care work in crisis situations ( Funk et al., 2019; Malaver-Fonseca et al., 2021). The following year, 2022, addresses contemporary issues such as the impact of automation in Latin America and the experiences of women teachers in neoliberal times. These studies contribute to the existing literature by highlighting the need to consider the gender implications of technological advances and educational environments ( Egana-delSol et al., 2022; Castelao-Huerta, 2022).

The period between 1974 and 2000 has seen a remarkable emergence and development of research in women’s studies in Latin America. During these decades, different cultures and research systems have played a crucial role in shaping the direction and focus of these studies. Initially, an incipient attention can be observed in 1974, which over time is transformed into an exponential growth of 99.5% in the annual production of scholarly articles on the subject. It is particularly relevant to highlight the years 2019, 2020, 2021, and 2022 as the culminating moments with the highest production, highlighting a constantly growing interest and recognition. This increase reflects not only the greater attention given to gender studies, but also the diversification of the topics addressed.

The influence of different cultures and research systems is evident in the variety of topics addressed over these years. From pioneering research on the experiences of gender violence faced by transgender women in different contexts ( Lanham et al., 2019) to more recent analyses examining the impact of automation in Latin America ( Egana-delSol et al., 2022), the diversity of approaches and concerns is evident. Similarly, the intersectionality of the studies is highlighted, with gender issues intertwined with cultural, economic, and political factors, highlighting the complexity of women’s experiences in the region.

Moreover, attention to issues such as gender equality in science ( Lopez-Aguirre, 2019), women’s political participation ( Funk et al., 2019), and the systematic review of the literature on the role of women in the care economy during the COVID-19 pandemic ( Malaver-Fonseca et al., 2021) shows how different cultures and research systems have contributed to the formation of a comprehensive and multidimensional perspective of women’s studies in Latin America. In this sense, the emerging research landscape reflects not only a quantitative increase, but also a qualitative evolution, in which the dynamic interaction between cultures and research systems has enriched the understanding of the complexity of the experiences and challenges faced by women in the region.

In reviewing research references on Latin America, the significant contributions of researchers such as Safa, Deere, Szafarz and Agier stand out, as well as the relevance of scientific journals such as World Development and Social Science and Medicine. These contributions reflect a diversity of approaches and key issues in gender studies in the region. Safa’s work on women’s social movements and Deere’s research on land tenure from a gender perspective offer critical visions that have enriched the understanding of social and economic dynamics in Latin America ( Safa, 1990, 1996; Deere & León, 2001, 2005). These perspectives, contextualized in their respective years of publication, reflect an ongoing commitment to exploring challenges and progress in the region.

Szafarz and Agier’s research in microfinance highlights the importance of addressing gender inequalities in access to financial resources, with their study revealing the phenomenon of the “glass ceiling” in lending to women in microfinance ( Agier & Szafarz, 2013). These findings have driven debates and policies in the field of financial inclusion, highlighting the need to take gender into account when designing economic strategies.

The journals World Development and Social Science and Medicine have provided important platforms for knowledge dissemination by addressing issues such as microfinance, health, and women’s decision-making. These studies, such as the analysis of the impact of the Bolsa Família program on women’s decisions ( De Brauw et al., 2014), contribute to a broader understanding of gender and health dynamics in Latin America and highlight the importance of an interdisciplinary approach. In the political sphere, research on women’s career trajectories and the impact of gender quotas and electoral laws provides valuable insights into women’s political participation in the region ( Escobar-Lemmon & Taylor-Robinson, 2009; Jones, 2009). These studies point to the need for inclusive policies to promote equitable representation in the political sphere.

The recognition of the United States and Canada for their contributions underscores the global relevance of gender studies in Latin America. Research on the role of the United States in promoting gender quotas and electoral laws in the region underscores its commitment to gender equality and women’s empowerment ( Jones, 2009). Similarly, Canada’s comparative study of cities in Latin America and the Caribbean on health disparities between men and women underscores its commitment to research on gender inequalities and their impact in the region ( Zunzunegui et al., 2009).

Over time, there has been a significant shift in the focus of studies on women in Latin America, as evidenced by the thematic evolution (see Figure 6). Initially, the focus was on the concept of the “household”, exploring the internal dynamics and gender inequalities in the domestic sphere, as exemplified by Fletschner’s research on rural women’s access to credit ( Fletschner, 2009). This pioneering approach highlighted how family structures affect women’s economic empowerment.

Over time, the concept of gender has gained prominence, highlighting its importance for gender equality and women’s entrepreneurship in Latin America ( Ruiz-Martínez, Kuschel & Pastor, 2021). Research highlights how understanding gender is fundamental to achieving equality in different sectors of society, as reflected in the analysis of the care practices of female teachers in a neoliberal academic environment ( Castelao-Huerta, 2023).

Gender equality is emerging as an essential concept in the fight for women’s rights, addressing everything from journalistic ethics in the face of gender violence to work inequalities during the COVID-19 pandemic ( Edo & Zurbano-Berenguer, 2019; Berniell et al., 2023). These studies highlight the need to promote gender equality in all spheres of life. On the other hand, in the area of violence against women, a study that examines the role of local governments in preventing violence during the pandemic stands out ( Lima, 2020). This work underscores the importance of addressing gender-based violence and highlights the crucial role of local authorities in protecting women’s rights in times of crisis.

The graphic representation of the themes, grouped into thematic clusters (see Figure 7), shows a focus on political participation, gender in institutions and women’s political representation (yellow), as well as an emphasis on feminisms, gender-based violence, women’s movements and resistance (red). These thematic groups highlight the diversity of aspects addressed by gender studies in Latin America, from the political sphere to feminist resistance and the fight against gender violence. Specific research, such as the analysis of the criminalization of political violence against women and decolonial portraits of the situation in Brazil, support these thematic approaches ( Sanín, 2022; Maso et al., 2022). This panorama highlights the breadth and complexity of issues related to gender studies in the region.

In terms of the analysis of the frequency and validity of keywords, within the second quadrant of our bibliometric analysis on women’s studies in Latin America, emerging concepts stand out that have become increasingly relevant in the scholarly field on this topic (see Figure 8). Three important keywords in this quadrant are: Neoliberalism, Violence against Women in Politics, and Women’s Empowerment. These concepts are of great importance today and are expected to remain relevant in the near future, as they reflect key challenges and opportunities for the advancement of rights and gender equality.

The concept of neoliberalism highlights the influence of this trend in contemporary society and, more specifically, in women’s experiences. A relevant study to understand this dynamic is research that examines the subtle mechanisms of violence experienced by women teachers in neoliberal times ( Castelao-Huerta, 2022). The article offers a critical perspective on how neoliberal policies and practices can perpetuate gender inequality and symbolic violence, highlighting the importance of analyzing broader social structures in the struggle for gender equality.

On the other hand, the issue of violence against women in politics has become increasingly relevant in the Latin American context (see Figure 7). A study of pioneering legislation in Bolivia that addresses violence against women in politics is presented as an important example. This work highlights the importance of promoting gender equality policies that protect women from violence and encourage their participation in the political sphere ( Castaño, 2022). By highlighting this issue, it seeks to ensure that women have equal opportunities and meaningful representation in political processes.

Furthermore, the concept of women’s empowerment is fundamental to the promotion of gender equality in Latin America. One study analyzes how women’s property ownership can influence the reduction of domestic violence in the region ( Gahramanov, Gaibulloev, & Younas, 2022). The results suggest that women’s economic empowerment through property can contribute to the reduction of gender-based violence. This highlights the importance of promoting policies and programs that strengthen women’s economic autonomy as an effective strategy to combat gender violence and promote gender equality.

The first quadrant, which includes emerging, prominent and consolidated concepts in research (see Figure 7), is of great importance in the study of gender. The term “gender” encompasses a wide range of research and theories that examine how gender characteristics and roles influence society. In this sense, the article entitled “Dynamic stereotypes about women and men in Latin America and the United States” offers an interesting perspective on gender stereotypes in different cultures ( Diekman, Eagly, Mladinic & Ferreira, 2005). This study analyzes gender stereotypes in Latin America and the United States and how these stereotypes can change and evolve in different contexts. The authors argue that gender stereotypes are dynamic and influenced by cultural and social factors. Through surveys and comparative analysis, the researchers show that gender stereotypes vary between Latin American and American cultures, suggesting the importance of considering cultural context in the study of gender.

The relevance of these concepts today lies in the need to understand and address persistent gender inequalities in our societies. By examining gender stereotypes, we can question and challenge long-held beliefs that limit people’s potential and opportunities based on their gender. Furthermore, by understanding how these stereotypes evolve and change across cultures, we can develop more effective strategies to promote gender equality globally.

### 4.2 Classification of keywords about Latin America according to their function

After analyzing the performance of the keywords in the present bibliometrics around thematic evolution, thematic clusters and recognition of frequency and validity, Table 1 is presented, which allows classifying the fundamental keywords in the context of studies of the women in Latin America based on their function.

**Table 1.** Classification of keywords according to their function. Own elaboration from Scopus and Web of Science.

Keyword	Associated tools	Applications	Characteristics
Neoliberalism	Economic analysis, Policy evaluation, Social inequality	Economic policies, Social reforms, Market dynamics	Market-oriented, Privatization, Individualism
Violence Against	Survey, Data collection, Intervention programs	Domestic violence, Sexual assault, Gender-based violence	Prevention, Support services, Legal frameworks
Women in Politics	Political representation, Electoral systems, Gender quotas	Political participation, Policymaking, Decision-making	Gender parity, Women’s empowerment, Inclusive governance
Women Empowerment	Education, Capacity building, Financial inclusion	Gender equality, Leadership development, Poverty reduction	Self-confidence, Agency, Autonomy
Femicide	Homicide investigations, Victim support, Legal frameworks	Gender-based killings, Intimate partner violence, Hate crimes	Gender-based violence, Justice, Advocacy
Gender	Gender analysis, Gender mainstreaming, Gender identity	Gender equality, Gender roles, Gender stereotypes	Social construction, Intersectionality, Gender justice

The previous classification is based on the emerging keywords in the subject of study, such as Neoliberalism, Violence Against, Women in Politics, Women Empowerment, Femicide and Gender, this classification is used as a key analysis instrument for future research to support its contributions from them.

### 4.3 Limitations

This research on women’s studies in Latin America is carried out based on the PRISMA-2020 methodology and uses the Scopus and Web of Science databases, some important limitations are found to take into account. First of all, it should be considered that the selection of the databases used may have generated a bias in the inclusion of studies, since there are other relevant sources of information in the field that might not have been considered; In addition, although tools such as Microsoft Excel ^®^ and VOSviewer ^®^ were used to analyze the bibliometric data, it is necessary to recognize that these programs have limitations in terms of their ability to capture and analyze the complexity and diversity of studies on women in Latin America.

A second important limitation lies in the choice of the bibliometric indicators used to assess the quantity, quality, and structure of the publications. Although these indicators can provide an overview of the scientific production, they do not necessarily capture the intrinsic quality of the studies nor do they fully reflect the thematic evolution and the contributions of the main referents in the field; Likewise, it is possible that the selection of keywords and the co-occurrence analysis do not cover all the relevant dimensions of women’s studies in Latin America, which could limit the representativeness and exhaustiveness of the results obtained. In summary, while this bibliometrics offers an overview of research in the field, it is essential to consider these limitations and complement it with more detailed and contextual analysis to obtain a full understanding of women’s studies in Latin America.

However, it is important to acknowledge the limitations of the statement regarding the implications of keywords and topic groups for practice. While these elements are said to have significant implications, the review does not delve into any specific topic that could provide substantial information to guide practice. The analysis’s lack of detailed development of specific themes can substantially limit practical application, as it requires a deeper and more contextualized understanding of key elements. To ensure that statements about the implications of keywords and clusters are supported by thorough analysis and applicable to specific situations, this gap needs to be addressed.

### 4.4 Investigative gaps

Considering the results obtained and the corresponding discussion and analysis, Table 2 is presented, which identifies a series of research gaps or conceptual gaps in the scientific field on the subject of gender studies in Latin America. This table provides a rationale and raises future questions that could address each of these identified gaps.

**Table 2.** Research gaps. Own elaboration from Scopus and Web of Science.

Category	Gaps	Justification	Questions to close the gaps
Thematic gaps	1. Studies on gender violence	There is a need for more comprehensive research that addresses the multiple manifestations, underlying factors, and prevention and response strategies for gender violence in Latin America.	What are the most effective strategies to prevent and address gender violence in different Latin American contexts?
	2. Representation of women in the media	Detailed research on the representation of women in the media in Latin America is limited, and a deeper understanding of gender stereotypes and their impact on society is required.	How does the representation of women in the media influence the construction of gender identities and gender inequalities in Latin America?
Geographic gaps	1. Comparative studies between countries	The lack of comparative studies between different Latin American countries makes it difficult to understand the similarities and differences in the experiences of women in the region.	What are the similarities and differences in the experiences of women in different Latin American countries?
	2. Research in rural and indigenous areas	The lack of focus on the experiences of women in rural areas and indigenous communities limits our understanding of the inequalities and specific challenges they face.	What are the main barriers to gender equality in rural areas and indigenous communities in Latin America?
Interdisciplinary gaps	1. Integration of gender approaches	Although gender studies have advanced in certain disciplines, there are still gaps in the integration of gender approaches in disciplines such as economics, politics and science.	How can gender analysis be better incorporated into research and practice in disciplines not traditionally associated with women's studies?
Temporary gaps	1. Limitations in longitudinal studies	Longitudinal studies are required that examine changes in the position of women in Latin America over time and its relationship with social, economic, and political factors.	What are the main drivers of changes in the position of women in Latin America over time? How have gender inequalities evolved in different historical contexts in the region? What policies and programs have had a significant impact in promoting gender equality in Latin America?

In this sense, it is observed that the research gaps, which are 7 in total, are categorized into thematic gaps, geographical gaps, interdisciplinary gaps and temporal gaps, which account for the heterogeneity of approaches that can be given to future studies that seek to fill these gaps.

### 4.5. Implications for research

**Figure 9.**
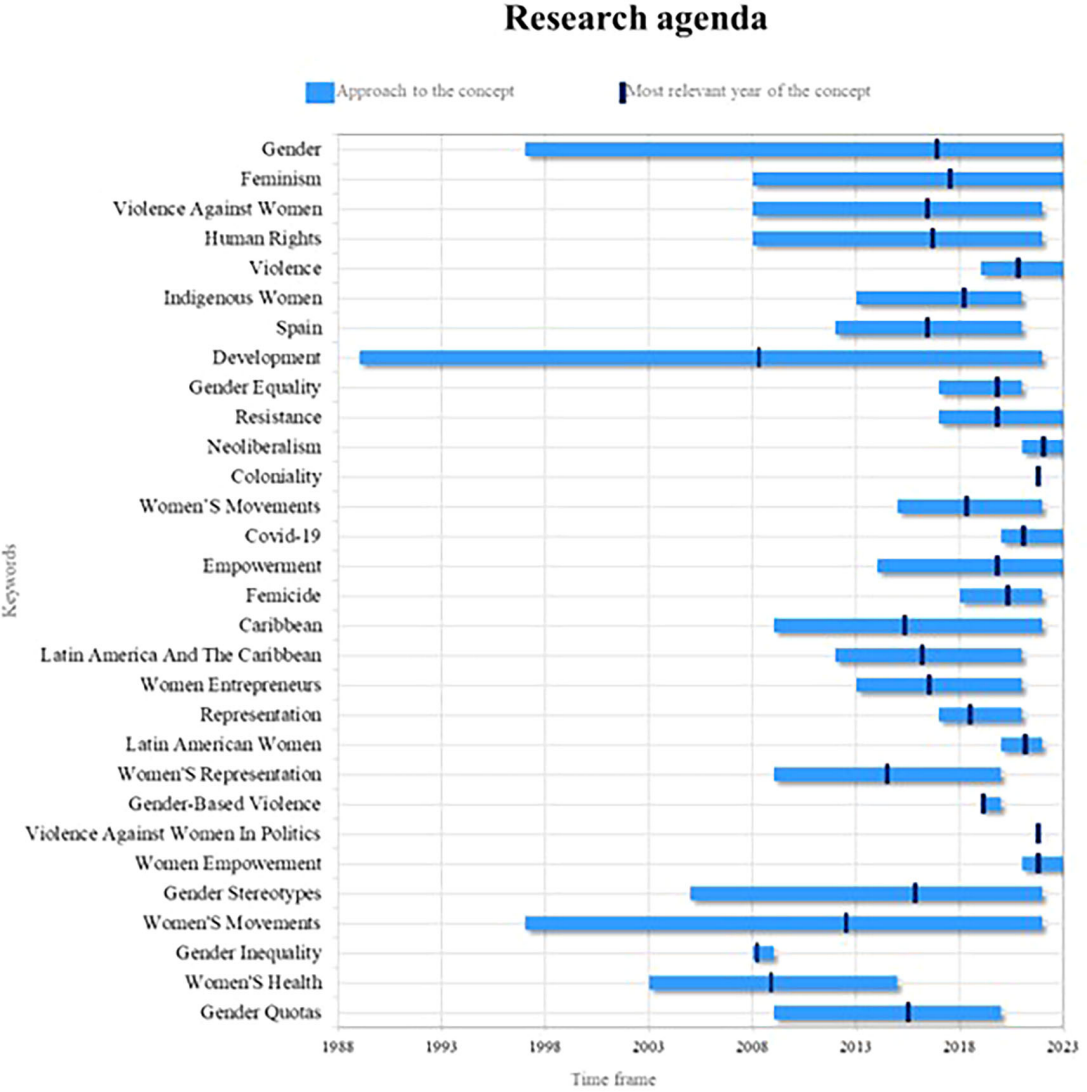
Research agenda. Own elaboration from Scopus and Web of Science.

Finally, the present bibliometric analysis on women’s studies proposes a fundamental research agenda based on the compilation of the outstanding concepts in the scientific literature in different periods of time, as observed in Figure 9, whose prominence is reflected in terms of consolidated, emerging and decreasing concepts and some others that, in light of the results and their analyses, account for valuable elements of analysis for future research.

In this sense, the key concepts in women’s studies in Latin America are gender, feminism and empowerment. At present, a greater awareness has been generated about the need to understand and analyze the power structures and gender inequalities in our society. For future research, it is crucial to explore how gender roles and power dynamics influence the lives of women in the region. In addition, it is possible to investigate the way in which women face and challenge established gender norms, and examine the impact of feminism in promoting gender equality and social transformation, it is also important to analyze how the empowerment of women is related to their participation in political, social and economic decision-making, and how this can contribute to development and gender justice in Latin America.

On the other hand, violence, resistance, neoliberalism, Covid-19 and the empowerment of women are emerging terms of great importance in the field of women’s studies in Latin America. For future research, it is crucial to investigate the various forms of violence faced by women in the region, from gender violence to structural and symbolic violence. Likewise, the resistance of women and feminist movements against these forms of violence and oppression must be analyzed. Regarding the current context, it must be analyzed how neoliberalism and the Covid-19 pandemic affect the lives of women in Latin America, in terms of socioeconomic inequality, access to health services, precarious work and gender violence. In addition, it is necessary to examine how the empowerment of women can be a strategy to face and overcome these challenges, promoting their autonomy, leadership and participation in the transformation of unequal power structures.

Finally, development, women’s movements, and gender stereotypes are highly relevant concepts in women’s studies in Latin America. Currently, it is essential to understand how development processes affect men and women differently, and how gender inequalities are perpetuated and reproduced in various areas of life. Future research can explore how women’s movements in the region challenge hegemonic development policies and practices, seeking more inclusive and equitable alternatives. Likewise, it should be investigated how gender stereotypes influence the lives of Latin American women, limiting their opportunities and perpetuating inequalities. Also, it is necessary to analyze how these stereotypes manifest themselves in different contexts, such as the workplace, education, politics and the media, and how they can be challenged and transformed to promote gender equality, as well as the empowerment of women in the region.

## 5. Practical implications

The present bibliometric analysis applied to women’s studies in Latin America reveals a significant thematic evolution in this area of research. Previously, the focus was on the study of households, but now it has been expanded to cover broader issues related to gender, gender equality, violence against women and feminism. This conceptual transformation reflects a change in understanding and interest in crucial issues for the advancement of women’s rights in the region.

One of the most outstanding findings is the formation of a thematic cluster in which the conceptual affinity between terms such as gender, political participation, institutions, gender perspective, politics and representation is identified. This suggests a growing intersection between gender and the political sphere, indicating a greater recognition of the importance of the gender perspective in public policy and decision-making.

In the same way, the analysis of frequency and validity of keywords shows emerging concepts such as neoliberalism, gender violence, women in politics, women’s empowerment and feminicide. These terms reflect current and urgent issues that require attention and action. Identification of these emerging concepts through bibliometrics provides a solid foundation for driving research and policy focused on addressing these issues and promoting meaningful social change.

In other words, the implications of these findings are numerous. First, they demonstrate the importance of strengthening academic research and knowledge production in the field of women’s studies in Latin America. In addition, they highlight the need to foster interdisciplinary collaboration between experts in gender, politics, sociology and other relevant disciplines to comprehensively address the identified problems.

Finally, these results underscore the importance of translating academic findings into effective public policies and concrete action programs. Bibliometrics provides a comprehensive and systematic view of the dominant trends and approaches in women’s studies, making it possible to identify areas of strength and weakness in research and policy implementation. Using this information to inform and guide political decisions can significantly contribute to promoting gender equality, preventing violence against women, and moving towards more just and inclusive societies in Latin America.

## 6. Conclusions

After carrying out a meticulous bibliometric analysis of studies on women in Latin America, significant results have been obtained that respond to the research questions posed. A notable increase in interest in this thematic area has been observed in recent years, with 2019, 2020, 2021 and 2022 being the most outstanding years in terms of the number of published studies. From this it is concluded that this increase reflects the importance and relevance given to the analysis of women and their role in Latin American society during this period.

Regarding the increase in scientific production on gender studies in Latin America, it is concluded that there is a clear pattern of exponential growth, this suggests that more and more researchers are focusing their efforts on addressing this issue, which demonstrates the importance and relevance attributed to the study of the problems and realities of women in the region.

When analyzing the most relevant referents in this area of research, the names of Safa and Deere stood out as prominent researchers, and the journals World Development and Social Science and Medicine as the main means of disseminating these studies. In addition, it was observed that the countries with the highest scientific production are the United States and Canada. It is concluded from this that there was international collaboration and cooperation in the advancement of research on gender studies in Latin America.

When examining the evolution of scientific production in this field, a notable change in the topics addressed is evident. In its beginnings, the literature focuses mainly on aspects related to housing, however, it has now broadened its focus to broader issues such as gender and equality. From this it is concluded that this change reflects greater awareness and attention to gender issues and their impact on Latin American society.

When analyzing the main thematic clusters, key issues such as gender, political participation, institutions, the gender perspective, politics and representation were identified. Thus, concluding that these clusters show a solid conceptual connection and highlight priority research areas in gender studies in Latin America.

Regarding rising and emerging keywords, the consolidation of fundamental concepts such as gender, which continues to be central in this area of research, is evident. Likewise, emerging concepts such as neoliberalism, gender violence, women’s political participation, female empowerment and feminicide were identified. From this it is concluded that these emerging concepts reflect the new concerns and challenges that are being addressed in the field of gender studies in Latin America.

## Ethics and consent

Ethical approval and consent were not required.

## Data Availability

The data availability statement for this study has been duly registered and archived in the Zenodo open data repository, which is recognized for its commitment to the accessibility and preservation of scientific data. The data and materials supported by this study are publicly available under a Creative Commons Attribution 4.0 International (CC BY 4.0) license and can be accessed at the following DOI link: https://doi.org/10.5281/zenodo.14841959 ( Valencia et al., 2024). Figshare: PRISMA checklist https://doi.org/10.5281/zenodo.14841959 ( Valencia et al., 2024). Data are available under the terms of the Creative Commons Attribution 4.0 International license (CC-BY 4.0).
