# Supplementary material for: Women's studies in the Latin American context: a bibliometric approach
Source: F1000Res. 2025 May 16;14:234. Originally published 2025 Feb 26. [Version 3] doi: 10.12688/f1000research.159117.3 (PMC12457888; doi:10.12688/f1000research.159117.3)
Supplement: Peer review history

# Reviewer report: Reviewer response for version 3

**DOI:** 10.5256/f1000research.182061.r410487

**Published:** 2025-09-30

**Authors:** Crystal Whetstone

**Affiliations:** 1 Bilkent University, Bilkent, Turkey

The authors have completed a bibliometric analysis of studies in Scopus and Web and Science journals on women's studies in Latin America, and they have been clear on the limitations that comes with this method and with the search term of Latin America rather than looking at individual countries. As I looked at the references I was wondering why certain key pieces I know from Latin American studies and women's political participation weren't here, but it's precisely because these studies didn't deal with Latin America but rather a few countries. I read with great interest the authors' findings, particularly the keywords for each year and the emerging themes in this area of research. It's clear that the study has been updated based on previous feedback, and I can't say anything further in terms of any changes that would make a significant difference to anything. This is an important contribution to having a 10,000m description of research on women's issues in Latin America, and I enjoyed the visuals provided in terms of getting an understanding. The only thing I might add is a bit of context about the emerging issues of femicide, neoliberalism, gender violence, political participation, etc, at least in defining at least femicide and neoliberalism, which could be unfamiliar terms to some. To conclude, this piece is an invaluable work for those interested in women's issues in Latin America.

Is the work clearly and accurately presented and does it cite the current literature?

Yes

If applicable, is the statistical analysis and its interpretation appropriate?

Not applicable

Are all the source data underlying the results available to ensure full reproducibility?

Yes

Is the study design appropriate and is the work technically sound?

Yes

Are the conclusions drawn adequately supported by the results?

Yes

Are sufficient details of methods and analysis provided to allow replication by others?

Yes

Reviewer Expertise:

Feminist comparative and international politics; women's political participation; gender-based violence; political motherhood

I confirm that I have read this submission and believe that I have an appropriate level of expertise to confirm that it is of an acceptable scientific standard.

# Reviewer report: Reviewer response for version 3

**DOI:** 10.5256/f1000research.182061.r402698

**Published:** 2025-09-23

**Authors:** Ömer Alkan

**Affiliations:** 1 Ataturk University, Erzurum, Turkey

I am happy having the opportunity to read and review the manuscript assigned to me, titled “Women's studies in the Latin American context: a bibliometric approach” submitted to the F1000Research.

 I have carefully reviewed this manuscript and below is my decision.

 The paper is well-suited for indexing in the F1000Research. All comments are well-improved the quality of paper. Good luck!

Is the work clearly and accurately presented and does it cite the current literature?

Yes

If applicable, is the statistical analysis and its interpretation appropriate?

Yes

Are all the source data underlying the results available to ensure full reproducibility?

Yes

Is the study design appropriate and is the work technically sound?

Yes

Are the conclusions drawn adequately supported by the results?

Yes

Are sufficient details of methods and analysis provided to allow replication by others?

Yes

Reviewer Expertise:

Statistical analysis

I confirm that I have read this submission and believe that I have an appropriate level of expertise to confirm that it is of an acceptable scientific standard.

# Reviewer report: Reviewer response for version 2

**DOI:** 10.5256/f1000research.179954.r378946

**Published:** 2025-05-09

**Authors:** Noelia Araújo-Vila

**Affiliations:** 1 University of Vigo, Ourense, Spain

The article provides a timely and relevant bibliometric analysis of women’s studies in the Latin American context. The abstract is well-structured and informative, clearly summarizing the research aim, methodology, results, and conclusions. However, the article lacks a dedicated theoretical framework section. The theoretical background is only briefly touched upon in the introduction, which is insufficient for a high-impact journal. A thorough review of key theories and conceptual developments in gender studies would strengthen the academic rigor and contextualize the bibliometric findings.

 The methodology is carefully designed, following PRISMA-2020 guidelines and applying robust bibliometric techniques using Scopus and Web of Science databases. Nevertheless, the search strategy could have been more exhaustive by including country-specific terms. The results are well-organized and visually supported by figures and tables, although some graphics could be more critically analyzed within the discussion. Conclusions are aligned with the findings but should address the study’s limitations more explicitly and propose concrete theoretical and practical implications. I recommend adding a detailed theoretical review section, improving the integration of figures into the discussion, and refining the analysis of research gaps.

Is the work clearly and accurately presented and does it cite the current literature?

Yes

If applicable, is the statistical analysis and its interpretation appropriate?

Yes

Are all the source data underlying the results available to ensure full reproducibility?

Yes

Is the study design appropriate and is the work technically sound?

Yes

Are the conclusions drawn adequately supported by the results?

Yes

Are sufficient details of methods and analysis provided to allow replication by others?

Yes

Reviewer Expertise:

Marketing, tourism, consumer behavior

I confirm that I have read this submission and believe that I have an appropriate level of expertise to confirm that it is of an acceptable scientific standard, however I have significant reservations, as outlined above.

# Response

**Published:** 2025-05-13

**Authors:** JHOANY ALEJANDRO VALENCIA ARIAS

We thank the reviewer for his kind and important contributions to the quality of this manuscript. The following comments are addressed:

 Comment: The article provides a timely and relevant bibliometric analysis of women’s studies in the Latin American context. The abstract is well-structured and informative, clearly summarizing the research aim, methodology, results, and conclusions. However, the article lacks a dedicated theoretical framework section. The theoretical background is only briefly touched upon in the introduction, which is insufficient for a high-impact journal. A thorough review of key theories and conceptual developments in gender studies would strengthen the academic rigor and contextualize the bibliometric findings.

 Response: Based on the reviewer's comments, a specific theoretical framework section was added, exploring the main conceptual and theoretical approaches to gender studies in Latin America, including feminist, intersectional, and neoliberal perspectives. This addition seeks to contextualize and strengthen the bibliometric findings presented in the article.

 Comment: The methodology is carefully designed, following PRISMA-2020 guidelines and applying robust bibliometric techniques using Scopus and Web of Science databases. Nevertheless, the search strategy could have been more exhaustive by including country-specific terms. The results are well-organized and visually supported by figures and tables, although some graphics could be more critically analyzed within the discussion. Conclusions are aligned with the findings but should address the study’s limitations more explicitly and propose concrete theoretical and practical implications. I recommend adding a detailed theoretical review section, improving the integration of figures into the discussion, and refining the analysis of research gaps.

 Response: The Limitations subsection was expanded to recognize the restriction on the use of the term "Latin America" ​​and its impact on the coverage of country studies. In addition, new subsections on theoretical and practical implications were incorporated, analyzing how the findings contribute to existing conceptual frameworks and public policymaking. Finally, the analysis of research gaps was refined, classifying them into thematic, geographic, interdisciplinary, and temporal categories to provide a clearer and more useful overview for future research.

# Reviewer report: Reviewer response for version 2

**DOI:** 10.5256/f1000research.179954.r375579

**Published:** 2025-05-07

**Authors:** Alma Angelica Villa-Rueda

**Affiliations:** 1 Universidad Autonoma de Baja California, Mexicali, Baja California, Mexico

Authors included all the recommendations.

Is the work clearly and accurately presented and does it cite the current literature?

Yes

If applicable, is the statistical analysis and its interpretation appropriate?

Not applicable

Are all the source data underlying the results available to ensure full reproducibility?

Yes

Is the study design appropriate and is the work technically sound?

Yes

Are the conclusions drawn adequately supported by the results?

Yes

Are sufficient details of methods and analysis provided to allow replication by others?

Partly

Reviewer Expertise:

Gender, sexuality, health.

I confirm that I have read this submission and believe that I have an appropriate level of expertise to confirm that it is of an acceptable scientific standard.

# Reviewer report: Reviewer response for version 1

**DOI:** 10.5256/f1000research.174801.r369009

**Published:** 2025-03-11

**Authors:** Alma Angelica Villa-Rueda

**Affiliations:** 1 Universidad Autonoma de Baja California, Mexicali, Baja California, Mexico

The present article aims to examine the trends in research on women’s studies in Latin America. 

 Introduction:

 The rationale presented at the introduction section is confusing. It looks like they are presenting articles as referents of the state of knowledge and to justify the present article, however, this style can confuse readers. In some point someone may think there are several aims. I recommend to improve the synthesis of knowledge for clarity purposes.

 Methods:

 It is not clear what authors mean by erroneous indexing, please explain.

 They state that they focused on systematic reviews, but this is not mentioned before. 

 It is not clear how authors decided to exclude texts deemed of marginal relevance to the study. Please clarify.

 There are other terms related to America Latina used in Mesh and Decs, like Mexico, Brasil etc.

 Overall this is a interesting article, even the results can look very descriptive more than analytical, they provide evidence for further research. The conclusion are well sustained based on their results. I recommend to expand the implications beyond academic research and be more specific with the processes of translating knowledge to public policy. I think this section is the most valuable of all articles.

Is the work clearly and accurately presented and does it cite the current literature?

Yes

If applicable, is the statistical analysis and its interpretation appropriate?

Not applicable

Are all the source data underlying the results available to ensure full reproducibility?

Yes

Is the study design appropriate and is the work technically sound?

Yes

Are the conclusions drawn adequately supported by the results?

Yes

Are sufficient details of methods and analysis provided to allow replication by others?

Partly

Reviewer Expertise:

Gender, sexuality, health.

I confirm that I have read this submission and believe that I have an appropriate level of expertise to confirm that it is of an acceptable scientific standard, however I have significant reservations, as outlined above.

# Response

**Published:** 2025-03-26

**Authors:** JHOANY ALEJANDRO VALENCIA ARIAS

We thank the reviewer for his valuable input. Below are the responses, point by point:

**Comment**: Introduction: The rationale presented at the introduction section is confusing. It looks like they are presenting articles as referents of the state of knowledge and to justify the present article, however, this style can confuse readers. In some point someone may think there are several aims. I recommend to improve the synthesis of knowledge for clarity purposes.

**Response**: The research objective was clearly defined and the justification expanded.

**Comment**: It is not clear what authors mean by erroneous indexing, please explain.

**Response**: Clarification was provided regarding misindexing.

**Comment**: They state that they focused on systematic reviews, but this is not mentioned before. 

**Response**: It was clarified that the removal of documents without full-text access applies to RSL and that the present study is a biometrics study.

**Comment**: It is not clear how authors decided to exclude texts deemed of marginal relevance to the study. Please clarify.

**Response**: The meaning of marginal relevance in the context of the study was clarified.

**Comment**: There are other terms related to America Latina used in Mesh and Decs, like Mexico, Brasil etc.

**Response**: This has been added as a limitation of the study, since expanding the search modifies the results and would require a new study.

**Comment**: Overall this is a interesting article, even the results can look very descriptive more than analytical, they provide evidence for further research. The conclusion are well sustained based on their results. I recommend to expand the implications beyond academic research and be more specific with the processes of translating knowledge to public policy. I think this section is the most valuable of all articles.

**Response**: The implications of the results were adjusted. These are discussed in more detail in the discussion section.
